# Pivot Teeth

**Published:** 1881-03

**Authors:** Charles L. Steel


### ARTICLE II.

### *Pivot Teeth.*

BY CHAS. L. STEEL, D. D. S.

In Harris' Dictionary of Medicine and Dental Surgery we find a pivot tooth defined as "an artificial tooth designed to be applied to the root of a natural tooth, by means of what is usually termed, a pivot, but more properly a dowel or tenon." He then adds, with what may seem sarcasm to many practitioners, especially young ones, " Also, a tooth thus applied," for there is certainly a great difference between a tooth *designed* to be " thus applied " and one " thus applied."

Pivot teeth are certainly a great advantage when only one or two teeth are missing, as they save the annoyance and possible injury of wearing a plate. Then, too, if we have a healthy root to which we can attach a tooth, we can give a more natural appearance than can be done with a plate. But on the other hand, they have their disadvantages, for some, especially those attached by wooden pivots, absorb the fluids of the mouth and soon become offensive.

Then, too, they are not very permanent. Though we may occasionally find one lasting through many years, yet it will be an exception, for the natural root, having lost its principal centre of nutrition, the pulp, is nourished only by the periosteum and so gradually becomes entirely dead, and, sooner or later, will be expelled by nature ; or, again, the root, becoming diseased, suppuration may set in, causing much trouble in the surrounding tissues, which, if not checked, may induce caries and necrosis of the bone.

Pivots are made either of wood or metal. If the former is used it should be of well seasoned and compressed young hickory, as this wood is stronger, tougher and more durable than any other. The fact of its being compressed allows it to absorb more moisture and therefore swell more and fit tighter. Various operators have different ways of inserting pivot teeth, and, strange to say, many still adhere to the old-fashioned style of using wood. The operators who advocate this practice, claim that the wood will absorb the moisture, swell and retain a tooth more firmly than will a metal pivot. But it seems to me that this same moisture, which is absorbed, will favor the decomposition of the wooden pivot, giving rise to offensive odors and causing the pivot to give way in a comparatively short time; then, again, the swelling of a wooden pivot has been known to split the root of a tooth; so I am almost inclined to fear that the dentists who use wood do so in order to avoid the slight extra labor of getting up a tooth with a metallic pivot. In Harris' Principles and Practice, a method of encasing a metallic pivot in wood is given. It seems to me that we here get the benefit of the objectionable features of the wooden pivot, without any of the advantages of the metal. The chief objection which has been urged against metallic pivots is that they wear the tooth substance with which they come in contact; this it seems to me can only become an object when the tooth is constantly removed from the mouth; but if the tooth is properly inserted with perfectly closed joints, it seems to me that its removal is perfectly unnecessary and, in fact, rather objectionable. Teeth attached by metallic pivots are, in my opinion, much nicer, more durable and more cleanly than those attached by wooden pivots.

Tomes, in his Dental Surgery, says: "If the canal is necessarily large, the wood pivot will be preferable." It is with great hesitancy that I presume to differ with such high authority, but *I* should say, if the canal is necessarily large, *always* use a *metallic* pivot, and for the following reasons: when the canal is very large the walls of the roots are proportionally thinner and therefore weaker; now the larger the canal the more wood there will be in the pivot and the more moisture it will absorb, therefore the more liable it will be to split the root, especially as the walls of the root are weaker. In the second place, the increased amount of wood present is more liable to give rise to an offensive odor. I suppose Tomes recommended wood because it would be difficult to adapt a metallic pivot to a large canal, but in a case of this sort, after carefully cleansing the canal and closing the apicial foramen with gold, the remaining portion may be filled with amalgam and in this a hole may be made of whatever size the operator may deem best. Some may say that the contact of the gold pivot with the amalgam might give rise to galvanic action, but I hardly think that would be found to give much trouble, especially in a pulpless root, and even if it were possible, the connection between the two metals may be broken by gutta percha in a way which I will speak of later on in this paper.

Superior central incisors are the teeth which are oftenest successfully pivoted, the lateral incisors sometimes being too small to pivot well; the cuspids usually being the last teeth to be lost, seldom require to have their roots pivoted. Bicuspid roots are sometimes pivoted, but unless the artificial crowns are quite firmly secured, the force of mastication is very liable to loosen them. Then, all bicuspid and molar roots are more liable to be expelled by the efforts of nature than the roots of the six anterior teeth. So noticeable is this fact that some authors have claimed that the anterior roots are endowed with a greater degree of vitality from their periosteum than the posterior. The lower incisors are very seldom pivoted as they are so small that they are difficult to pivot securely and then one would not be very much missed if lost. The first step in pivoting teeth is the preparation of the root. We will suppose a tooth to have been broken off, the remaining portion of the crown should be cut off with small saws or the excising forceps; in using the forceps care should be exercised not to take hold of too large a portion of the tooth at once as this produces a jar, which not only causes unnecessary pain to the patient, but may also induce so much irritation that destructive inflammation will be set up. When the crown has been cut off near the gum, a half round file or, better still, a corrundum wheel in the dental engine, may be used and the root cut off a little below the free margin of the gum, and so as to cause it to present a concave surface. The canal in the root should now be nicely cleaned out and if there is any disease about the root, it should be perfectly cured before any attempt is made to attach the artificial tooth. We should never attach any artificial crown to any but a *perfectly* healthy root. The canal should now be reamed out for about one-fourth of an inch of its length, until its diameter is about one-sixteenth of an inch, and should be nicely filled from the apex to the point to which the pivot is to extend. If the pivot is to be of wood, we must now select a tooth of the proper size, shape and color, and of a kind manufactured specially for pivoting with a hole for the pivot opposite the canal in the root; this tooth is now ground until it fits accurately against the exposed surface of the root. A piece of young hickory wood should now be taken and trimmed until it is almost, but not quite small enough to go in the canal; this should now be passed through a smooth hole in some hard substance, in order to compress its fibres—one end of this wood is now wedged into the hole in the artificial crown and cut off as far from the tooth as the pivot is to extend into the root canal. A very good, though expensive, and troublesome method is as follow :

After preparing the root as above described, take a piece of gold wire, either square or round, not quite so large as the cavity in the root; burnish around this a piece of thin platinum, forming a tube of platinum in which the gold wire fits perfectly; withdraw the wire and solder the platinum together; replace the wire and insert the tube with pivot enclosed; now fill around the tube with cohesive gold and allow the gold to cover the whole surface of the root, thereby protecting the root from decay, and holding in the tube; now select a thin plate tooth of the proper size, shape and color, grind it until it fits the surface of the gold cap and surrounding gum exactly, then solder a backing to it; now take an impression of the gold covering the root, get up a die and counter and swage a platina cap to accurately fit the exposed surface of gold; in this platina cap, cut a hole through which the pivot may pass; place the pivot, cap and tooth in position; now press soft wax against the tooth and platinum so as to hold them together; withdraw the pivot and tooth from the mouth, invest them in plaster and asbestos, and solder them. After finishing this up properly it is ready for insertion in the mouth. The pivot must fit in the tube as accurately as a ground joint, or the tooth will not be sufficiently firm. The pivot may be made of two pieces of an alloy of gold and platina which is quite springy; after fitting this to the tube and attaching the tooth as before, the two parts of which the pivot is composed should be slightly sprung apart and when inserted in the tube, they will act as a constant spring, holding the tooth tightly in place. This is unquestionably a nice operation and especially adapted to cases where the nerve canals have been much enlarged, but if it is very troublesome work, and unfortunately we seldom find patients willing to pay the fee which it is worth, so, for all practical purposes, I think the following method answers every purpose and is certainly much simpler. Prepare the root and ream out the canal as before. Now take a piece of platina or gold wire a shade smaller than the canal and fit it as high up in the root as it is desirable that the pivot should go; allow the wire to extend about one-eighth of an inch below the root; now while the pin is still in take an *accurate* impression of the exposed surface of the root and one or two of the adjoining teeth; the pin will be drawn away with the impression and from this must be secured a model of plaster and asbestos. When this is separated from the impression the pin must remain in the model; now take a piece of very thin platinum, punch a hole in it to allow the pin to go through and then burnish this platinum over that part of the model which corresponds to the exposed surface of the root in the mouth; cut off the projecting portion of the pin. A suitable thin plate tooth having been selected, grind it to fit closely against the root and surrounding gum, retain it in position with wax, invest the whole and solder the pin, plate and backing together. When this is nicely dressed up it is ready for insertion in the mouth. To do this, warm the pin and coat it with Hill's stopping, warm this until it is quite soft and then press the pin firmly up until the tooth is in the proper position, and when the gutta-percha is hard, the surplus may be cut away. A few notches should be cut in the pivot and one or two grooves in the canal in order to assist the gutta-percha to retain the tooth in position. The gutta-percha makes all joints perfect and, being covered by the free margin of the gum, it will preserve the root from decay almost indefinitely. If the pin, plate and backing are all of platinum the soldering may be done with pure gold, and I think this preferable. In making the cap for this case I burnish the platinum over that part of the model corresponding to the exposed surface of the root in the mouth. This can often be done satisfactorily, but for greater accuracy, it is always best, in making these caps to swage them on a metallic die.

The Bonwill Crown is applied as follows: porcelain teeth are made specially to be applied in this way, with a hole of considerable size opening on the palatine surfaces of single rooted teeth and on the grinding surfaces of teeth posterior to the canines. The root is prepared as in the cases previously described: the tooth applicable to the case in hand is then selected and ground to accurately fit the root; a gold or platina wire is now fitted through the whole in the artificial tooth and extending up into the root; the tooth is now withdrawn and the pin fastened in the canal with some filling material; the tooth is now put in position, the wire extending into the cavity in the tooth; this is now attached by means of some filling material; Dr. Bonwill, I believe, recommend amalgam. In attaching bicuspid and molar crowns, a wire is fixed in all the roots and these wires bent together, forming something of an arch in the cavity of the tooth, helping to retain the crown more securely, when the filling material is inserted.

A favorite method with some operators, amongst whom may be mentioned Dr. Foster and Dr. Marshall H. Webb, is to solder a wire to the pins of a suitably prepared thin plate tooth, place the tooth and wire in position and retain them in position by building around the wire with cohesive foil; the palatine surface of the tooth is then contoured with cohesive foil. While I do not know that they come under the head of what is usually meant by a pivot tooth, yet I do not think this paper would be complete without some mention of the extremely nice operation resorted to by some of our profession, of inserting one or more artificial crowns, where the roots have been lost, by attaching them to the adjacent teeth, thus doing away with the necessity for a plate covering the mouth. To suppose an extreme case, let us say that the four superior incisors, roots and all were gone. We would first take an impression of the part and swage a plate to fit the ridge to be occupied by the teeth. Solder the teeth to be inserted to this plate and have extensions from the backing of the laterals to go into cavities cut in the cuspids; put this in position in the mouth and fill the cavities which contain the extensions, with gold. This, when well performed, makes a very nice operation.

